# Supplementary material for: Cardiotoxic profiles of CAR-T therapy and bispecific T-cell engagers in hematological cancers
Source: Commun Med (Lond). 2024 Jun 13;4:116. doi: 10.1038/s43856-024-00540-9 (PMC11176393; doi:10.1038/s43856-024-00540-9)
Supplement: Supplementary file 2 — Supplementary Materials [file 43856_2024_540_MOESM2_ESM.docx]

**Cardiotoxic Profiles of CAR-T Therapy and Bispecific T-Cell Engagers in Hematological Cancers**

Supplementary Material

Badri Karthikeyan^1,2^; Sunitha Shyam Sunder^2^; Igor Puzanov^3^; Scott H. Olejniczak^4^;

Saraswati Pokharel^2^; Umesh C. Sharma^1^

^1^Department of Medicine, Division of Cardiology, Jacobs School of Medicine and Biomedical Sciences, Buffalo, NY, USA, 14203

^2^Department of Pathology and Laboratory Medicine, Roswell Park Comprehensive Cancer Center, Buffalo, NY, USA, 14203

^3^Department of Medicine, Division of Oncology, Roswell Park Comprehensive Cancer Center, Buffalo, NY, USA, 14203

^4^Department of Immunology, Roswell Park Comprehensive Cancer Center, Buffalo, NY, USA, 14203

Supplementary Table 1. Information about the CAR-T therapies and bispecific T-cell engagers investigated.

| ***CAR-T Therapy*** | | | |
| --- | --- | --- | --- |
| **Generic Name** | **Brand Name** | **Target** | **FDA Approval Date** |
| Tisagenlecleucel | Kymriah | CD19 | August 30, 2017 |
| Axicabtagene ciloleucel | Yescarta | CD19 | October 18, 2017 |
| Brexucabtagene autoleucel | Tecartus | CD19 | July 24, 2020 |
| Lisocabtagene maraleucel | Breyanzi | CD19 | February 5, 2021 |
| Idecabtagene vicleucel | Abecma | BCMA | March 26, 2021 |
| Ciltacabtagene autoleucel | Carvykti | BCMA | February 28, 2022 |
| ***Bispecific T-Cell Engager*** | | | |
| **Generic Name** | **Brand Name** | **Target** | **FDA Approval Date** |
| Blinatumomab | Blincyto | CD19, CD3 | December 3, 2014 |

FDA: Food and Drug Administration

Supplementary Table 2. A 2 × 2 contingency table for calculating reporting odds ratios between two drug categories for a given reaction.

|  | **Number of reports containing a given adverse reaction** | **Number of reports that does not contain a given adverse reaction** |
| --- | --- | --- |
| **Number of reports containing a drug category (e.g. CAR-T Therapy)** | A | B |
| **Number of reports containing another drug category (e.g. Blinatumomab)** | C | D |

$$Reporting odds ratio \left( \mathrm{ROR} \right)=\frac{A/C}{B/D}=\frac{\mathrm{AD}}{\mathrm{BC}}$$

**Supplementary Table 3: Information components (IC) calculated for tisagenlecleucel (N_D_ = 2,233) for the given adverse reactions.**

| **Reaction Name** | **N_A_** | **N_AD_** | **IC** | **IC_025_** |
| --- | --- | --- | --- | --- |
| ***Inflammatory/Effusive Events*** | | | | |
| **C-Reactive Protein Increased** | **25,169** | **51** | **4.441** | **3.976** |
| **Capillary Leak Syndrome** | **1,206** | **13** | **4.517** | **3.578** |
| Myocarditis | 28,484 | 1 | -0.803 | -4.586 |
| Pericarditis | 21,552 | 1 | -0.487 | -4.270 |
| **Pericardial Effusion** | **17,867** | **12** | **2.773** | **1.795** |
| Cardiac Tamponade | 2,897 | 0 | -0.517 | -10.840 |
| ***Arrhythmic Events*** | | | | |
| **Tachycardia** | **202,122** | **89** | **2.527** | **2.176** |
| **Atrial Fibrillation** | **79,948** | **15** | **1.266** | **0.395** |
| Atrial Flutter | 6,773 | 2 | 1.317 | -1.276 |
| Electrocardiogram QT Prolonged | 27,465 | 5 | 1.114 | -0.449 |
| Ventricular Tachycardia/Ventricular Fibrillation | 27,573 | 6 | 1.350 | -0.065 |
| **Cardiac Arrest** | **75,180** | **14** | **1.252** | **0.349** |
| ***Hemodynamic Events*** | | | | |
| **Hypotension** | **268,722** | **307** | **3.909** | **3.720** |
| **Sinus Tachycardia** | **14,075** | **9** | **2.619** | **1.480** |
| ***Cardiomyopathy-Related Events*** | | | | |
| Stress Cardiomyopathy | 2,700 | 1 | 1.098 | -2.685 |
| **Ejection Fraction Decreased** | **10,708** | **5** | **2.085** | **0.523** |
| **Cardiac Failure** | **130,976** | **22** | **1.081** | **0.366** |
| Cardiogenic Shock | 7,138 | 1 | 0.541 | -3.242 |

IC_025_ > 0 is an indicator for significant adverse reaction signals; N_D_ = number of reports containing a given medication in VigiBase; N_A_ = number of reports containing a given adverse reaction in VigiBase; N_AD_ = number of reports containing a given drug-adverse reaction pair in VigiBase.

**Supplementary Table 4: Information components (IC) calculated for axicabtagene ciloleucel (N_D_ = 3,035) for the given adverse reactions.**

| **Reaction Name** | **N_A_** | **N_AD_** | **IC** | **IC_025_** |
| --- | --- | --- | --- | --- |
| ***Inflammatory/Effusive Events*** | | | | |
| **C-Reactive Protein Increased** | **25,169** | **18** | **2.604** | **1.812** |
| Capillary Leak Syndrome | 1,206 | 1 | 1.270 | -2.513 |
| Myocarditis | 28,484 | 1 | -1.171 | -4.954 |
| Pericarditis | 21,552 | 1 | -0.836 | -4.619 |
| **Pericardial Effusion** | **17,867** | **6** | **1.496** | **0.081** |
| Cardiac Tamponade | 2,897 | 0 | -0.665 | -10.989 |
| ***Arrhythmic Events*** | | | | |
| **Tachycardia** | **202,122** | **80** | **1.944** | **1.573** |
| **Atrial Fibrillation** | **79,948** | **28** | **1.732** | **1.101** |
| **Atrial Flutter** | **6,773** | **5** | **2.215** | **0.653** |
| Electrocardiogram QT Prolonged | 27,465 | 4 | 0.458 | -1.307 |
| **Ventricular Tachycardia/Ventricular Fibrillation** | **27,573** | **8** | **1.371** | **0.159** |
| **Cardiac Arrest** | **75,180** | **16** | **1.027** | **0.185** |
| ***Hemodynamic Events*** | | | | |
| **Hypotension** | **268,722** | **152** | **2.463** | **2.195** |
| **Sinus Tachycardia** | **14,075** | **8** | **2.145** | **0.932** |
| ***Cardiomyopathy-Related Events*** | | | | |
| **Stress Cardiomyopathy** | **2,700** | **5** | **2.831** | **1.269** |
| **Ejection Fraction Decreased** | **10,708** | **6** | **2.039** | **0.624** |
| Cardiac Failure | 130,976 | 8 | -0.748 | -1.961 |
| Cardiogenic Shock | 7,138 | 3 | 1.519 | -0.550 |

IC_025_ > 0 is an indicator for significant adverse reaction signals; N_D_ = number of reports containing a given medication in VigiBase; N_A_ = number of reports containing a given adverse reaction in VigiBase; N_AD_ = number of reports containing a given drug-adverse reaction pair in VigiBase.

**Supplementary Table 5: Information components (IC) calculated for brexucabtagene autoleucel (N_D_ = 170) for the given adverse reactions.**

| **Reaction Name** | **N_A_** | **N_AD_** | **IC** | **IC_025_** |
| --- | --- | --- | --- | --- |
| ***Inflammatory/Effusive Events*** | | | | |
| C-Reactive Protein Increased | 25,169 | 1 | 1.223 | -2.560 |
| Capillary Leak Syndrome | 1,206 | 0 | -0.020 | -10.343 |
| Myocarditis | 28,484 | 0 | -0.403 | -10.727 |
| Pericarditis | 21,552 | 0 | -0.315 | -10.639 |
| Pericardial Effusion | 17,867 | 0 | -0.266 | -10.589 |
| Cardiac Tamponade | 2,897 | 0 | -0.047 | -10.370 |
| ***Arrhythmic Events*** | | | | |
| **Tachycardia** | **202,122** | **5** | **1.742** | **0.180** |
| Atrial Fibrillation | 79,948 | 2 | 1.392 | -1.201 |
| Atrial Flutter | 6,773 | 1 | 1.478 | -2.305 |
| Electrocardiogram QT Prolonged | 27,465 | 0 | -0.391 | -10.714 |
| Ventricular Tachycardia/Ventricular Fibrillation | 27,573 | 1 | 1.193 | -2.590 |
| Cardiac Arrest | 75,180 | 0 | -0.888 | -11.212 |
| ***Hemodynamic Events*** | | | | |
| **Hypotension** | **268,722** | **9** | **2.233** | **1.094** |
| Sinus Tachycardia | 14,075 | 1 | 1.372 | -2.411 |
| ***Cardiomyopathy-Related Events*** | | | | |
| Stress Cardiomyopathy | 2,700 | 0 | -0.043 | -10.367 |
| Ejection Fraction Decreased | 10,708 | 0 | -0.165 | -10.489 |
| Cardiac Failure | 130,976 | 0 | -1.347 | -11.671 |
| Cardiogenic Shock | 7,138 | 0 | -0.112 | -10.436 |

IC_025_ > 0 is an indicator for significant adverse reaction signals; N_D_ = number of reports containing a given medication in VigiBase; N_A_ = number of reports containing a given adverse reaction in VigiBase; N_AD_ = number of reports containing a given drug-adverse reaction pair in VigiBase.

**Supplementary Table 6: Information components (IC) calculated for lisocabtagene maraleucel (N_D_ = 82) for the given adverse reactions.**

| **Reaction Name** | **N_A_** | **N_AD_** | **IC** | **IC_025_** |
| --- | --- | --- | --- | --- |
| ***Inflammatory/Effusive Events*** | | | | |
| C-Reactive Protein Increased | 25,169 | 0 | -0.186 | -10.510 |
| Capillary Leak Syndrome | 1,206 | 0 | -0.009 | -10.333 |
| Myocarditis | 28,484 | 0 | -0.209 | -10.532 |
| Pericarditis | 21,552 | 1 | 1.424 | -2.359 |
| Pericardial Effusion | 17,867 | 0 | -0.134 | -10.458 |
| Cardiac Tamponade | 2,897 | 0 | -0.023 | -10.346 |
| ***Arrhythmic Events*** | | | | |
| Tachycardia | 202,122 | 0 | -1.073 | -11.397 |
| Atrial Fibrillation | 79,948 | 0 | -0.523 | -10.846 |
| Atrial Flutter | 6,773 | 0 | -0.052 | -10.376 |
| Electrocardiogram QT Prolonged | 27,465 | 0 | -0.202 | -10.525 |
| Ventricular Tachycardia/Ventricular Fibrillation | 27,573 | 0 | -0.202 | -10.526 |
| Cardiac Arrest | 75,180 | 1 | 1.089 | -2.694 |
| ***Hemodynamic Events*** | | | | |
| Hypotension | 268,722 | 0 | -1.303 | -11.627 |
| Sinus Tachycardia | 14,075 | 0 | -0.107 | -10.431 |
| ***Cardiomyopathy-Related Events*** | | | | |
| Stress Cardiomyopathy | 2,700 | 0 | -0.021 | -10.345 |
| Ejection Fraction Decreased | 10,708 | 0 | -0.082 | -10.406 |
| Cardiac Failure | 130,976 | 0 | -0.803 | -11.127 |
| Cardiogenic Shock | 7,138 | 0 | -0.055 | -10.379 |

IC_025_ > 0 is an indicator for significant adverse reaction signals; N_D_ = number of reports containing a given medication in VigiBase; N_A_ = number of reports containing a given adverse reaction in VigiBase; N_AD_ = number of reports containing a given drug-adverse reaction pair in VigiBase.

**Supplementary Table 7: Information components (IC) calculated for idecabtagene vicleucel (N_D_ = 54) for the given adverse reactions.**

| **Reaction Name** | **N_A_** | **N_AD_** | **IC** | **IC_025_** |
| --- | --- | --- | --- | --- |
| ***Inflammatory/Effusive Events*** | | | | |
| C-Reactive Protein Increased | 25,169 | 0 | -0.125 | -10.449 |
| Capillary Leak Syndrome | 1,206 | 0 | -0.006 | -10.330 |
| Myocarditis | 28,484 | 0 | -0.141 | -10.464 |
| Pericarditis | 21,552 | 0 | -0.108 | -10.431 |
| Pericardial Effusion | 17,867 | 0 | -0.090 | -10.414 |
| Cardiac Tamponade | 2,897 | 0 | -0.015 | -10.339 |
| ***Arrhythmic Events*** | | | | |
| Tachycardia | 202,122 | 0 | -0.788 | -11.112 |
| Atrial Fibrillation | 79,948 | 0 | -0.365 | -10.688 |
| Atrial Flutter | 6,773 | 0 | -0.035 | -10.358 |
| Electrocardiogram QT Prolonged | 27,465 | 0 | -0.136 | -10.460 |
| Ventricular Tachycardia/Ventricular Fibrillation | 27,573 | 0 | -0.136 | -10.460 |
| Cardiac Arrest | 75,180 | 0 | -0.345 | -10.669 |
| ***Hemodynamic Events*** | | | | |
| Hypotension | 268,722 | 1 | 0.610 | -3.174 |
| Sinus Tachycardia | 14,075 | 0 | -0.071 | -10.395 |
| ***Cardiomyopathy-Related Events*** | | | | |
| Stress Cardiomyopathy | 2,700 | 0 | -0.014 | -10.338 |
| Ejection Fraction Decreased | 10,708 | 0 | -0.054 | -10.378 |
| Cardiac Failure | 130,976 | 0 | -0.576 | -10.899 |
| Cardiogenic Shock | 7,138 | 0 | -0.037 | -10.360 |

IC_025_ > 0 is an indicator for significant adverse reaction signals; N_D_ = number of reports containing a given medication in VigiBase; N_A_ = number of reports containing a given adverse reaction in VigiBase; N_AD_ = number of reports containing a given drug-adverse reaction pair in VigiBase.

**Supplementary Table 8: Information components (IC) calculated for ciltacabtagene autoleucel (N_D_ = 2) for the given adverse reactions.**

| **Reaction Name** | **N_A_** | **N_AD_** | **IC** | **IC_025_** |
| --- | --- | --- | --- | --- |
| ***Inflammatory/Effusive Events*** | | | | |
| C-Reactive Protein Increased | 25,169 | 0 | -0.005 | -10.329 |
| Capillary Leak Syndrome | 1,206 | 0 | 0.000 | -10.324 |
| Myocarditis | 28,484 | 0 | -0.005 | -10.329 |
| Pericarditis | 21,552 | 0 | -0.004 | -10.328 |
| Pericardial Effusion | 17,867 | 0 | -0.003 | -10.327 |
| Cardiac Tamponade | 2,897 | 0 | -0.001 | -10.324 |
| ***Arrhythmic Events*** | | | | |
| Tachycardia | 202,122 | 0 | -0.038 | -10.362 |
| Atrial Fibrillation | 79,948 | 0 | -0.015 | -10.339 |
| Atrial Flutter | 6,773 | 0 | -0.001 | -10.325 |
| Electrocardiogram QT Prolonged | 27,465 | 0 | -0.005 | -10.329 |
| Ventricular Tachycardia/Ventricular Fibrillation | 27,573 | 0 | -0.005 | -10.329 |
| Cardiac Arrest | 75,180 | 0 | -0.014 | -10.338 |
| ***Hemodynamic Events*** | | | | |
| Hypotension | 268,722 | 0 | -0.051 | -10.374 |
| Sinus Tachycardia | 14,075 | 0 | -0.003 | -10.326 |
| ***Cardiomyopathy-Related Events*** | | | | |
| Stress Cardiomyopathy | 2,700 | 0 | -0.001 | -10.324 |
| Ejection Fraction Decreased | 10,708 | 0 | -0.002 | -10.326 |
| Cardiac Failure | 130,976 | 0 | -0.026 | -10.350 |
| Cardiogenic Shock | 7,138 | 1 | 1.584 | -2.200 |

IC_025_ > 0 is an indicator for significant adverse reaction signals; N_D_ = number of reports containing a given medication in VigiBase; N_A_ = number of reports containing a given adverse reaction in VigiBase; N_AD_ = number of reports containing a given drug-adverse reaction pair in VigiBase.

Supplementary Table 9. Clinical characteristics of patients treated with CAR-T therapy for diffuse large B-cell lymphoma reporting atrial fibrillation in VigiBase clinical study reports (N = 11).

| **Characteristic** | **Number of Patients (% of Total)** |
| --- | --- |
| **Gender** |  |
| Male | 7 (63.6%) |
| Female | 4 (36.4%) |
| **Age Group** |  |
| 45-64 years | 4 (36.4%) |
| 65-74 years | 5 (45.5%) |
| ≥75 years | 2 (18.2%) |
| **Region Reporting** |  |
| Americas | 8 (72.7%) |
| Europe | 3 (27.3%) |
| **Reporting Year** |  |
| 2021 | 4 (36.4%) |
| 2020 | 4 (36.4%) |
| 2019 | 2 (18.2%) |
| 2018 | 1 (9.1%) |
| **CAR-T Medications Used** |  |
| Tisagenlecleucel | 8 (72.7%) |
| Axicabtagene ciloleucel | 3 (27.3%) |
| **Chemotherapy Agents Used** |  |
| Cyclophosphamide | 7 (63.6%) |
| Fludarabine | 7 (63.6%) |
| **Treatments for Cytokine Release Syndrome Used** |  |
| Tocilizumab | 1 (9.1%) |
| **Cardiovascular Medications Used** |  |
| Beta blockers | 1 (9.1%) |
| Diltiazem | 1 (9.1%) |
| **Seriousness of Case** |  |
| Seriousness | 11 (100%) |
| Death | 5 (45.5%) |
| Life threatening | 1 (9.1%) |
| Caused/prolonged hospitalization | 3 (27.3%) |
| Other | 2 (18.2%) |
| **Concurrent Cardiovascular Adverse Reactions** |  |
| Hypotension | 5 (45.5%) |
| Tachycardia | 2 (18.2%) |
| Cardiac failure | 1 (9.1%) |
| Ejection fraction decreased | 1 (9.1%) |
