# Supplementary material for: Cardiotoxic profiles of CAR-T therapy and bispecific T-cell engagers in hematological cancers
Source: Commun Med (Lond). 2024 Jun 13;4:116. doi: 10.1038/s43856-024-00540-9 (PMC11176393; doi:10.1038/s43856-024-00540-9)
Supplement: Supplementary file 3 — Description of Additional Supplementary Files [file 43856_2024_540_MOESM3_ESM.pdf]

Description of Additional Supplementary Files

File name- Supplementary Data 1

File description- Clinical characteristics of the pediatric patients

File name- Supplementary Data 2

File description- Reaction reports by year
